# 

**Published:** 1880-08

**Authors:** 


					﻿AUGUST, 1880.
TWENTY-SEVENTH YEAR.
|||» HALL’S '
Journal of Health.
E. H. GIBBS, A.M., M.D., Editor,
No. 141 EIGHTH STREET (near Broadway) NEW YORK.
| Terms, $1.50 a Year, or $1.00 for Eight Months. Postage paid.
Entered as Second-Class matter in the Post-office in New York.
				

